# Supplementary material for: Non-thermal emission in gap-mode plasmon photoluminescence
Source: Nat Commun. 2024 May 25;15:4468. doi: 10.1038/s41467-024-48928-4 (PMC11127923; doi:10.1038/s41467-024-48928-4)
Supplement: Supplementary file 1 — Supplementary Information [file 41467_2024_48928_MOESM1_ESM.pdf]

Supplementary Information: Non-Thermal Emission in Gap-Mode Plasmon Photoluminescence

Robert Lemasters,* Manoj Manjare, Ryan Freeman, Feng Wang, Luka Guy Pierce, Gordon Hua, Sergei Urazhdin, and Hayk Harutyunyan*

Department of Physics, Emory University, Atlanta, Georgia 30322, USA

E-mail: robertdlemasters@gmail.com; hayk.harutyunyan@emory.edu

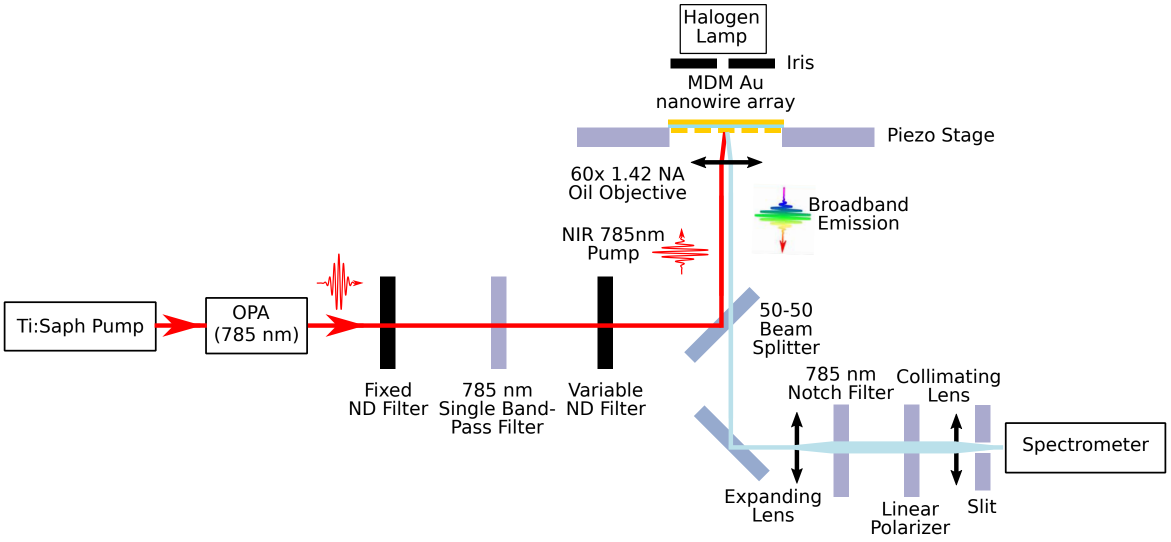

Supplementary Figure 1: **NPL and optical transmittance experimental setup.** Detail is given in the main text.

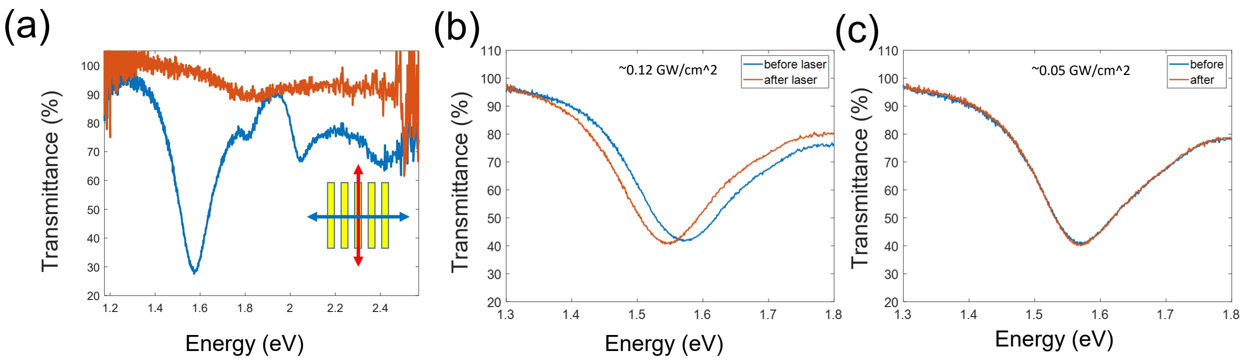

Supplementary Figure 2: **Polarization dependence and laser damage threshold.** (a) Polarization dependence of plasmon resonance and correlation with grating nanowire geometry. (b) Before and after laser irradiation for spectral shifting of plasmon mode due to high peak power laser induced restructuring of nanowire. (c) Before and after laser irradiation for confirmation of safe laser fluence/peak powers.

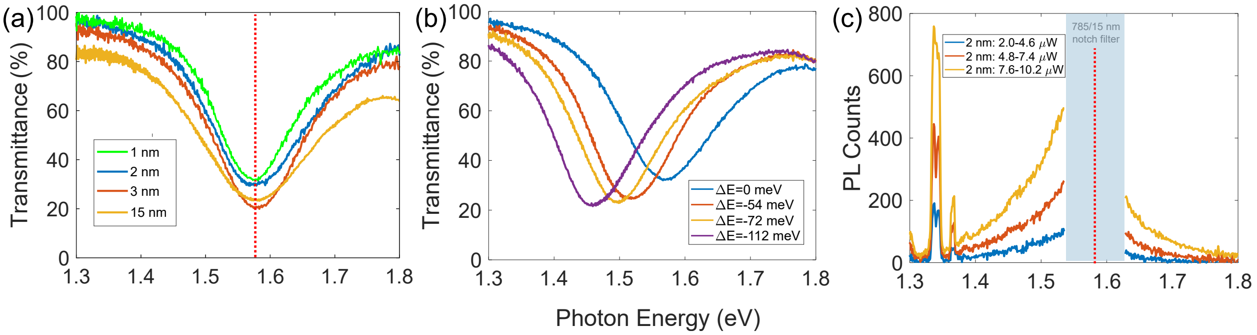

Supplementary Figure 3: **Experimental variables.** (a) Matching of plasmon resonances to the excitation laser energy (red dashed) among SiO_2 thicknesses of 1, 2, 3 and 15 nm. (b) Detuning of plasmon resonances from the excitation laser energy (red dashed) for a SiO_2 thicknesses of 2 nm. (c) NPL emission counts from MDM structures with 2 nm SiO_2 at varying non-overlapping laser power fluences.

Modeling and Simulations

Mathematical Analysis of NPL Lineshapes

An arbitrary optical emission signal, $\phi(\omega)$, which has an unknown nonlinear response corresponding to an excitation power of I can be expressed as

$$\phi(\omega) \propto I^{p(\omega)}, \tag{1}$$

where $p(\omega)$ is the power law exponent (PLE) for a signal at frequency, ω. As discussed in the main text, many nonlinear signals may be present and can overlap with one another, especially at increasingly higher excitation powers. In these cases, the extracted effective PLE for a particular frequency represents an average of all the nonlinear contributions at that same frequency. For some reference value, ϕ_0, at a power, I_0, the most generic way to extrapolate the PLE corresponding to each frequency, ω, is given by[1]

$$p(\omega) = \frac{\mathrm{d}}{\mathrm{d}\log\left(\frac{I}{I_0}\right)} \log \frac{\phi(\omega)}{\phi_0(\omega)}. \tag{2}$$

Each individual mechanism will have a characteristic PLE which can be extracted numerically or analytically using Eq. 2. The individual various PL mechanisms suggested and referenced in the literature, their transition diagrams and their corresponding emission spectrum, $\phi(\epsilon)$, and PLE, $p(\epsilon)$, are shown in Supplementary Figure 4. The individual state variables (if any) which contribute to the PLE lineshape are varied to demonstrate roughly the trends and overall character. These state variables may be altered experimentally.

Multi-Photon PL

In general, the response of the electrons may be written in terms of the polarizability, $\vec{P}(t)$, as

$$\vec{P}(t) = \epsilon_0 \left(\chi^{(1)} \vec{E}(t) + \chi^{(2)} \vec{E}^2(t) + \chi^{(3)} \vec{E}^3(t) + ... \right), \tag{3}$$

where $\chi^{(n)}$ is the nth order susceptibility tensor. In traditional nonlinear optical phenomenon the power scaling exponent is typically equal to the order number of the term in the expansion in eq. 3. This has the convenient relationship to the photon participation number. An example is given for second harmonic generation (SHG) for which two photons with frequency, Ω, combine to produce another photon with frequency equal to 2Ω. Typically, this results in a very narrow emission range with exponent, $p(\omega = 2\Omega) = 2$. However, two-photon absorption (TPA) also has a value of $p = 2$, but it's emission range is wider. The emission ranges is based on the local photonic density of states, $\rho(\omega)$, and is related to the spectrum, $\phi(\omega)$. This nonlinear character is present over the entire range, i.e., $p(\omega) = 2$. For a general multi-photon absorption process involving n photons, the emission spectrum will be given as, $\phi(\omega) = \rho(\omega) I^{p(\omega)}$, with $p(\omega) = n$. This mechanism is shown in Supplementary Figure 4a.

Raman Scattering

This Raman scattering section follows a derivation given by[2] . For a molecule positioned at $\vec{r}_0$ near a metal nanostructure, an incident field, $\vec{E}_0$, induces a dipole moment of

$$\vec{p}(\omega_{\mathrm{R}}) = \alpha(\omega_{\mathrm{R}}, \omega) \left[\vec{E}_0(\omega_{\mathrm{R}}, \omega) + \vec{E}_s(\omega_0, \omega) \right] \tag{4}$$

where $\omega_R = \omega \pm \omega_{\mathrm{vib}}$ with ω_{vib} being the molecule's vibrational frequency, and $\vec{E}_s$ is the scattered field from the molecule interacting with the nanostructures. α is the polarizability which is modulated at a frequency of ω_{vib} which causes a frequency mixing process between the incident and scattered fields, i.e. $\alpha(\omega_{\mathrm{R}}, \omega)$. The scattered electric field will scale linearly with the incident field. However, the incident field is enhanced by the nanostructures, and so we write the scattered field as

$$\vec{E}_s = f_1(\omega) \vec{E}_0, \tag{5}$$

where $f_1(\omega)$ measures the plasmonic enhancement of the field. It is also convenient to represent the response of the system using the method of Green's functions. This method provides a way to represent the relevant differential equations governing a system in terms of an impulse-response analysis. The derivation of this is given elsewhere.[3] With this in mind, we can estimate the far-field of the scattered light from the system as

$$\vec{E}(\vec{r}_\infty, \omega_{\mathrm{R}}) = \frac{\omega_{\mathrm{R}}^2}{\epsilon_0 c^2} \vec{G}(\vec{r}_\infty, \omega_{\mathrm{R}}) \left| \vec{p}(\omega_{\mathrm{R}}) \right| = \frac{\omega_{\mathrm{R}}^2}{\epsilon_0 c^2} \left[\vec{G}_0(\vec{r}_\infty, \omega_{\mathrm{R}}) + \vec{G}_s(\vec{r}_\infty, \omega_{\mathrm{R}}) \right] \left| \vec{p}(\omega_{\mathrm{R}}) \right|, \tag{6}$$

where again we have broken the Green's functions into two parts analogous to the dipole moment. Similarly as what has been done for $\vec{E}_s$, the scattered field represented by the Green's function is enhanced by a factor of

$$\vec{G}_s = f_2(\omega_{\mathrm{R}}) \vec{G}_0. \tag{7}$$

f_1 and f_2 differ is that one corresponds to the dipole moment enhancement of the molecule, and the other corresponds to out coupling from the plasmon mode. This is analogous to modulation of signals in analog antennas, where a signal is mixed with a strong emitter to transmit to the far-field. The intensity at the far-field scales as $I \propto \left|\vec{E}\right|^2$. Plugging everything together, we have

$$I(\vec{r}_\infty, \omega_\mathrm{R}) = \frac{\omega_\mathrm{R}^4}{\epsilon_0^2 c^4} \left| [1 + f_2(\omega_\mathrm{R})]\, \vec{G}_0(\vec{r}_\infty, \omega_\mathrm{R})\, [1 + f_1(\omega)] \right|^2 I_0(\vec{r}_\infty, \omega_\mathrm{R}). \tag{8}$$

From this it is clear that the far-field intensity of the enhanced Raman signal, ω_R, scales as

$$I(\vec{r}_\infty, \omega_\mathrm{R}) \propto \big|\, [1 + f_2(\omega_\mathrm{R})]\, [1 + f_1(\omega)] \,\big|^2 \tag{9}$$

$$\simeq \left|f_2(\omega_\mathrm{R})\right|^2 \left|f_1(\omega)\right|^2, \quad \text{for} \quad f_1, f_2 \gg 1 \quad \text{(i.e., strong enhancement).} \tag{10}$$

This result is what is refered to when stating that Raman signals scale as the 4th power of the electric field. However, closer analysis shows that this doesn't imply a quadratic scaling of the far-field intensity. This is due to the subtlety of what element of the molecule/nanostructure system is enhanced. In this case, it is the dipole moment of the system to the incoming field at ω, and the out coupling strength of the field at ω_R. i.e., scattering, not coherence. This mechanism is shown in Supplementary Figure 4b.

Hot-Raman Scattering

The Raman scattering mechanism can be extended to when applied to an elevated electron distribution. This is modeled by correcting the Raman signal with a factor which accounts for a temperature dependent electron-phonon scattering as

$$\phi(\omega) \propto E \left|1 + f_B(\hbar\omega_{inc} - \hbar\omega, T_e)\right| \cdot \frac{\left|\omega_{inc} - \omega\right| \tau_s}{1 + \tau_s^2 \left(\omega_{inc} - \omega\right)^2} \tag{11}$$

where f_B is the Boson distribution, $\hbar\omega_{inc}$ is the incoming photon excitation energy and τ_s is the effective scattering time. The modification comes from additional electronic scattering off of impurities described by the so-called "dirty metal" model.[4,5] A similar analysis starting with Eq. 2 gives[1]

$$p(\omega) = 1 + \frac{\hbar\omega - \hbar\omega_{inc}}{ak_B T_e}\left[1 + f_B(\hbar\omega, T_e)\right]. \tag{12}$$

This mechanism is shown in Supplementary Figure 4c.

Hot-Electron Intraband PL

In the assumption of neglecting bandstructure constraints, i.e. allowed diagonal intraband transitions, the electron-hole recombination and resulting emission spectrum is given as[1,6]

$$\phi(\omega) \propto \int_{-\infty}^{\infty} f_e(\epsilon, T_e)\rho(\omega) f_h(\epsilon - \hbar\omega, T_e)\mathrm{d}\epsilon = \hbar\omega\rho(\omega) f_B(\hbar\omega, T_e), \tag{13}$$

where f_e is the electronic Fermi distribution, $f_h = 1 - f_e$ is the hole Fermi distribution and ρ is the photonic density of states. The effective electronic temperature, T_e, is related to the excitation power, P, as

$$T_e = T_{e,0}\left(\frac{I}{I_0}\right)^{1/a}, \tag{14}$$

where the $_0$ subscripts the reference (room) temperature and reference power, respectively, and a is the effective thermal power coefficient typically taken to be $a = 2$ for metals. Plugging Eq. 13 into Eq. 2 yields

$$p(\omega) = \frac{\hbar\omega}{ak_B T_e}\left[1 + f_B(\hbar\omega, T_e)\right], \tag{15}$$

which for $k_B T_e \gg \hbar\omega$ reduces to

$$p(\omega) \approx \frac{\hbar\omega}{a k_B T_e}. \tag{16}$$

This mechanism is shown in Supplementary Figure 4d.

Non-Fermi Electron Intraband PL

Starting with the same assumptions of allowed intraband transitions, the estimate for non-Fermi type electron-hole recombination and emission spectrum can be modeled by simply replacing a Fermi with an athermal non-Fermi distribution. The spectrum is found by modifying Eq. 13 with a non-Fermi type distribution, giving

$$\phi(\omega) \propto \int_{-\infty}^{\infty} f_{\mathrm{nf},e}(\epsilon, T_0)\rho(\omega) f_{\mathrm{nf},h}(\epsilon - \hbar\omega, T_0)\ \mathrm{d}\epsilon. \tag{17}$$

The details of mathematical specifics of this emission is outlined in the main text. This mechanism is shown in Supplementary Figure 4e. A summary of the hybrid Fermi and non-Fermi electronic distribution parameters for the various experimental configurations in the main text is given in Table 1.

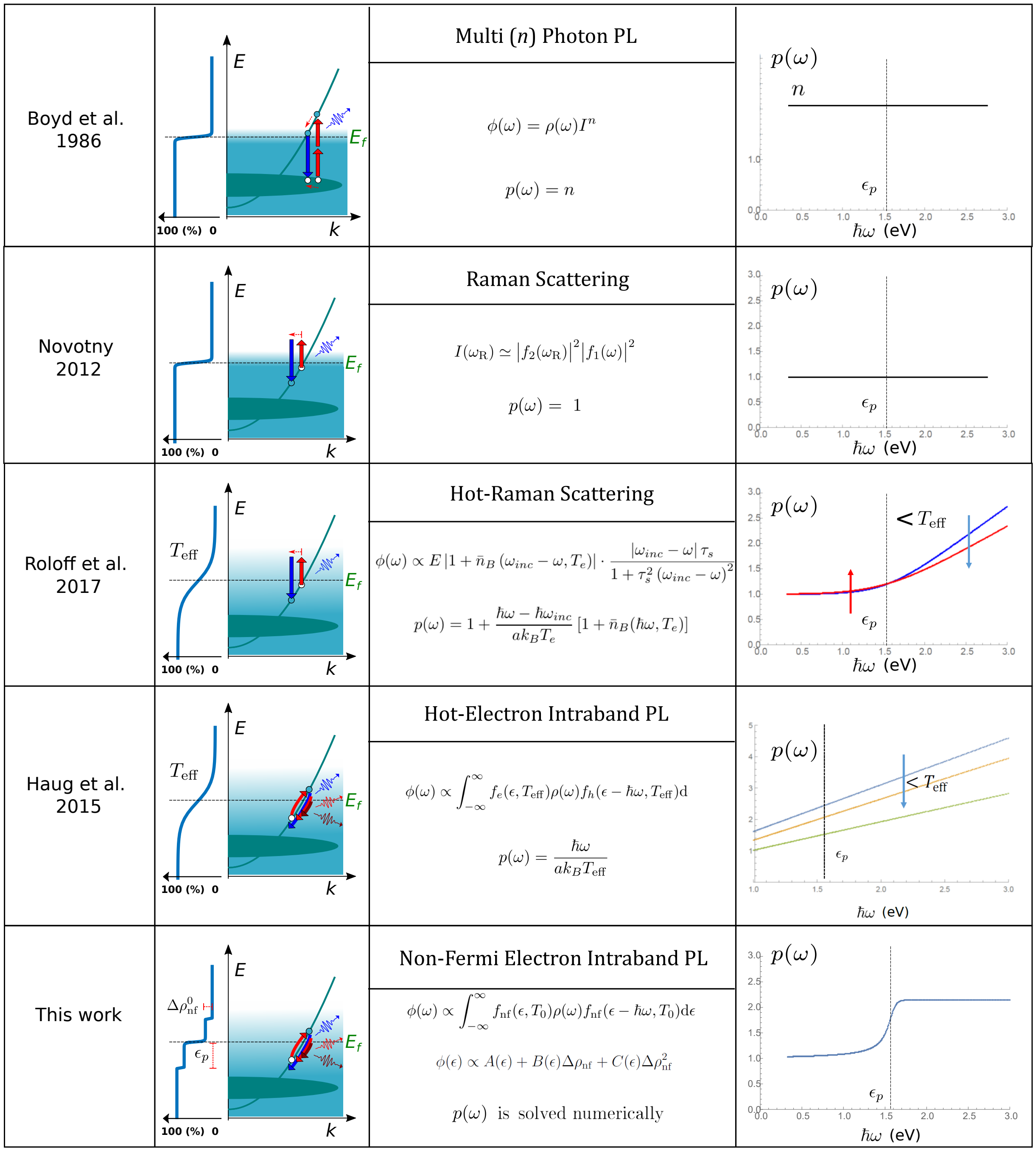

Supplementary Figure 4: **Nonlinear emission mechanisms.** Various nonlinear light emission mechanisms from different works, the expected spectra, $\phi(\omega)$ and their expected power law lineshape, $p(\omega)$. The referenced works from top to bottom are,[7],[2],[16] and this presented work.

FDTD Simulations

Modeling and simulation of the MDM structures was performed using Lumerical FDTD EM solver software. FDTD simulations of the electric field enhancement calculated at 785 nm for gap spacer thicknesses of 2, 3 and 15 nm SiO_2 are shown in Supplementary Figures. 5(a-c). The confinement of the electric field is clearly seen to increase with decreasing gap thickness and the relative field enhancement compared to the free space light reaches up to ~ 70. Additionally, the electric field gradients are clearly seen to sharpen as the spacer layer is decreased. For reference, experimental plasmon resonance matching spectra for dielectric gap spacer thicknesses of 2, 3 and 15 nm of SiO_2 are shown in Supplementary Figure 5(d).

It is crucial to determine whether this effect is due to the imposed characteristic wavevector (field gradients) or simply an enhancement effect due to the strongly enhanced electric fields. As noted in Supplementary Figures 5(a-c), the electric field dramatically increases in the gap mode for small thicknesses. To investigate this, the plasmon modes are detuned from the laser energy to make it slightly off-resonance. FDTD simulations of the electric field enhancement calculated at 785 nm for detuned modes of $\Delta E = 0$, -54 & -112 meV on a 3 nm SiO_2 spacer are shown in Supplementary Figures 5(e-g). The field enhancement drops accordingly for increasing detuning while the field gradients are seen to essentially remain stationary in the geometry. For reference, experimental plasmon resonance detuning spectra for $\Delta E = 0$, -54, -72 and -112 meV on 3 nm of SiO_2 are shown in Supplementary Figure 5(h).

Table 1: **Hybrid carrier distribution fitting parameters.** Summary of fitting parameters of hybrid hot-electron and non-Fermi electron distributions for reproducing PLE's. Physical intuition.

PLE lineshape fitting summary		State variables	Physical intuition
Fixed resonance/ varied gap (0 meV)	1 nm	$\Delta\rho^0_{\mathrm{nf}} = 0.0072 - 0.018$<br>$T_{\mathrm{eff}} = 5596 - 6838$ K<br>$\alpha = 1/200$	Both field strength and field gradients are enhanced with decreasing gap geometry, increasing $\Delta\rho^0_{\mathrm{nf}}$ and T_{eff}.
	2 nm	$\Delta\rho^0_{\mathrm{nf}} = 0.0024 - 0.006$<br>$T_{\mathrm{eff}} = 2906 - 3551$ K<br>$\alpha = 1/900$	
	3 nm	$\Delta\rho^0_{\mathrm{nf}} = 0.00036 - 0.0009$<br>$T_{\mathrm{eff}} = 1918 - 2344$ K<br>$\alpha = 1/15$	
	15 nm	$\Delta\rho^0_{\mathrm{nf}} = 4.8 - 12 \times 10^{-6}$<br>$T_{\mathrm{eff}} = 1918 - 2344$ K<br>$\alpha = 1/400$	
Fixed gap/ varied resonance (2 nm)	0 meV	$\Delta\rho^0_{\mathrm{nf}} = 0.0012 - 0.003$<br>$T_{\mathrm{eff}} = 2853 - 3487$ K<br>$\alpha = 1/300$	Effective wavevector is fixed by geometry and given field gradients. ($\Delta\rho^0_{\mathrm{nf}}$ =constant). Only transient T_{eff} from field enhancement significantly alters the signal.
	-54 meV	$\Delta\rho^0_{\mathrm{nf}} = 0.0012 - 0.003$<br>$T_{\mathrm{eff}} = 2140 - 2615$ K<br>$\alpha = 1/40$	
	-72 meV	$\Delta\rho^0_{\mathrm{nf}} = 0.0012 - 0.003$<br>$T_{\mathrm{eff}} = 1783 - 2149$ K<br>$\alpha = 1/8$	
	-112 meV	$\Delta\rho^0_{\mathrm{nf}} = 0.0012 - 0.003$<br>$T_{\mathrm{eff}} = 1426 - 1743$ K<br>$\alpha = 5/8$	
Fixed gap and resonance/ varied fluence ranges (2 nm/0 meV)	2.0-4.6 μJ	$\Delta\rho^0_{\mathrm{nf}} = 0.0016 - 0.0029$<br>$T_{\mathrm{eff}} = 2659 - 3028$ K<br>$\alpha = 1/900$	The spectral weight is fixed and only $\Delta\rho^0_{\mathrm{nf}}$ is needed for input as this fixes T_{eff} as described in the main text.
	4.8-7.4 μJ	$\Delta\rho^0_{\mathrm{nf}} = 0.003 - 0.0043$<br>$T_{\mathrm{eff}} = 3051 - 3301$ K<br>$\alpha = 1/900$	
	7.6-10.2 μJ	$\Delta\rho^0_{\mathrm{nf}} = 0.0044 - 0.0057$<br>$T_{\mathrm{eff}} = 3318 - 3511$ K<br>$\alpha = 1/900$	

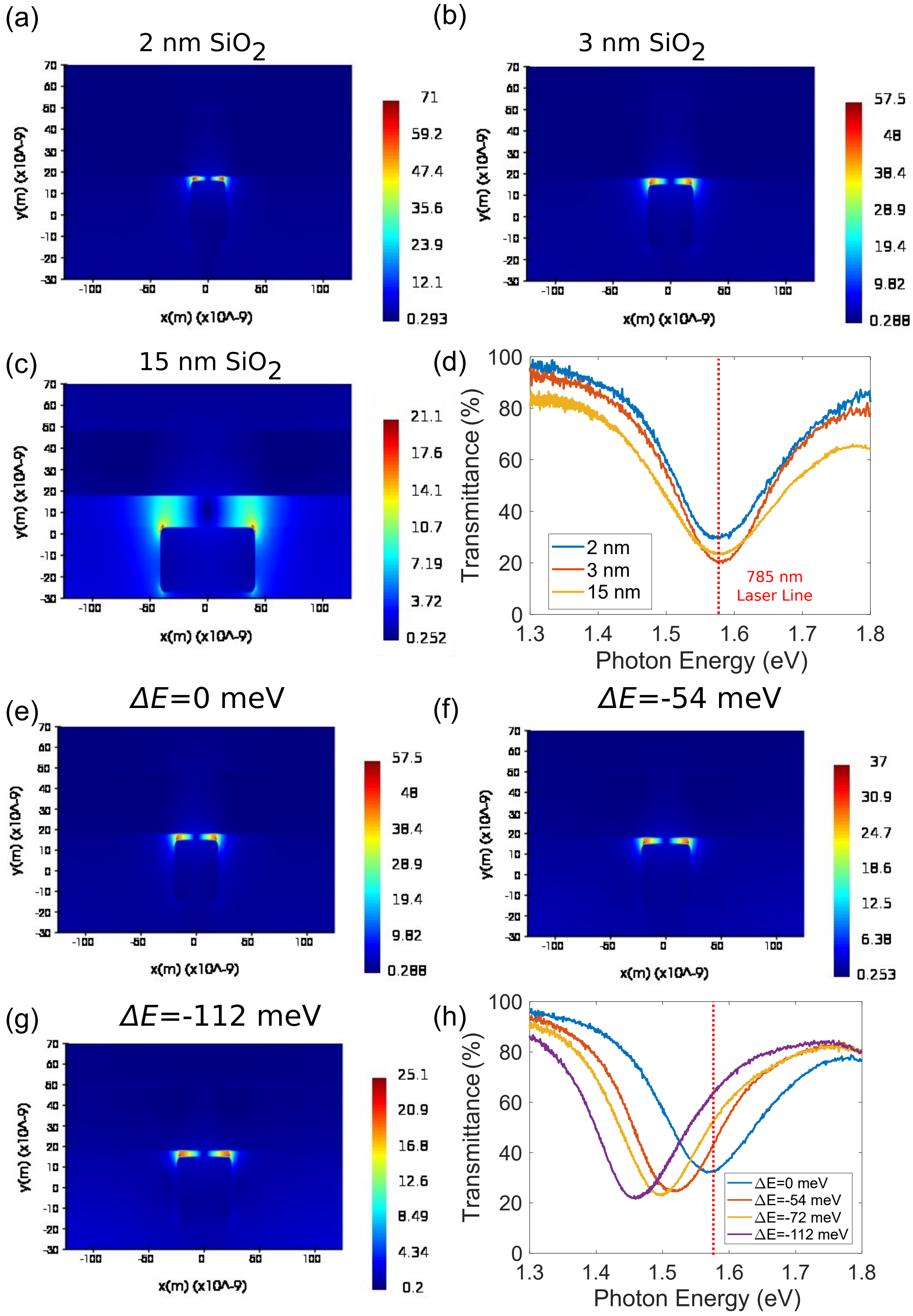

Supplementary Figure 5: **Finite-difference time-domain (FDTD) simulations.** (a-c) FDTD simulations of field enhancement distribution at 785 nm for fixed plasmon energy and varied gap size. (d) Corresponding experimental fixed plasmon energy and varied gap size transmittance. (e-g) FDTD simulations of field enhancement distribution at 785 nm for fixed gap size and varied plasmon energy. (h) Corresponding experimental fixed gap size and varied plasmon energy transmittance.

Dynamic Temperature Modeling

We model a time varying Fermi-Dirac distribution,

$$f(\epsilon, T) = \frac{1}{e^{\frac{\epsilon}{kT}} + 1}, \quad \epsilon = \hbar\omega, \tag{18}$$

which emits light during the thermal relaxation by first defining a time-varying temperature, $T(t)$, which exponentially decreases to the ambient thermal temperature, T_{amb}, through dissipation to the lattice on the order of the electron-phonon coupling time, $\tau_{\mathrm{e-ph}}$, as

$$T(t) = (T_{\mathrm{max}} - T_{\mathrm{amb}})\, e^{-t/\tau_{\mathrm{e-ph}}} + T_{\mathrm{amb}}, \tag{19}$$

where T_{max} is the maximum temperature the electron gas gets shortly after plasmon absorption, and $\tau_{\mathrm{e-ph}} \sim 1$ ps. This profile is shown in Supplementary Figure 6a. Since we are only concerned with the thermalized Fermi hot-electron distribution, we can calculate the spectrum to be

$$\phi(\epsilon) \propto \int_{-\infty}^{\infty} f(\epsilon', T)\rho(\epsilon) f(\epsilon - \epsilon', T)\mathrm{d}\epsilon' = \rho(\epsilon)\frac{\epsilon}{e^{\frac{\epsilon}{kT}} - 1}. \tag{20}$$

If we consider emission contributions from the thermalizing distribution at each time step up to the electron-phonon coupling timescale, the spectrum of the time-dependent temperature distribution is given by

$$\phi(\omega) \propto \frac{1}{A} \sum_{i}^{\tau_{\mathrm{e-ph}}/\Delta t} \rho(\epsilon)\frac{\epsilon}{e^{\frac{\epsilon}{kT(t_i)}} - 1} \tag{21}$$

where $A = \tau_{\mathrm{e-ph}}/\Delta t$ is a normalization constant and Δt is the time step interval chosen for the calculation to define $t_i = 0 : \Delta t : \tau_{\mathrm{e-ph}}$. We use the 2 nm thermal ranges extracted from our fits and use $\Delta t = 5$ fs. The extracted PLE for a time varying temperature and that of a constant temperature are shown in Supplementary Figure 6b.

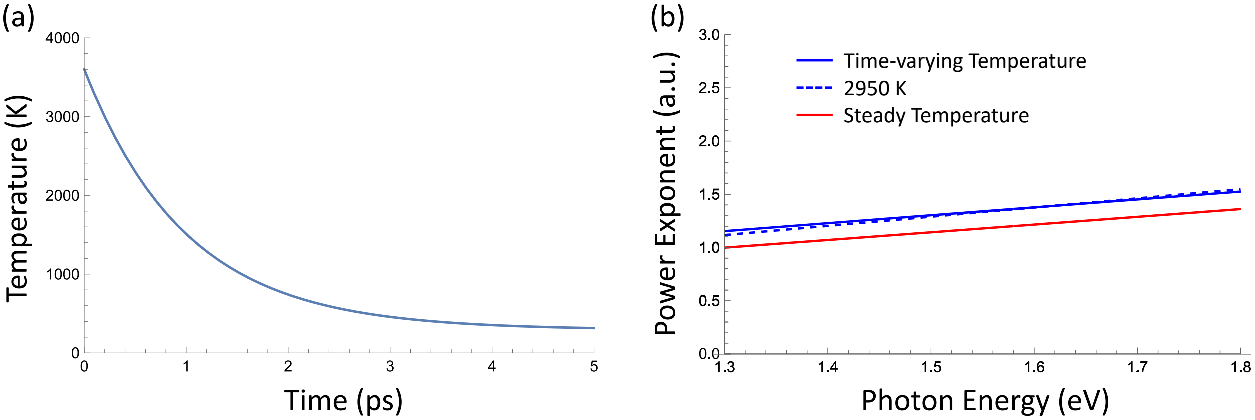

Supplementary Figure 6: **Time-varying temperature photoluminescence (PL).** (a) Exponentially decaying temperature towards ambient values. (b) PLE lineshapes calculated with 2 nm gap thermal parameters for an exponentially decaying temperature and a constant temperature. Dashed line shows the fitting for the effective temperature of the time varying PLE lineshape.

The dashed line shows the fitting for the effective temperature of the time varying temperature PLE lineshape. It is apparent that the PLE is well-modeled with the effective temperature approach. It is also clear that the PLE does not have any features which could be mistakenly identified as non-Fermi (see Supplementary Figure 4); it merely produces that which is expected for a Fermi distribution at an effective temperature. Therefore, a more robust and treatment of the temperature cannot reproduce the experimental PLE lineshapes of nanogap NPL without invoking a non-Fermi term.

Linear Emission Spectra Fitting

Direct fitting parameters extracted from PLE lineshapes are used to calculate the linear emission spectra. Fitting to a 2 nm spacer thickness on resonance, gives a fit of $\Gamma = 0.083$ eV and $\epsilon_R = 1.58$ eV. In this case, this is captured in the photonic density of states approximated by the plasmonic scattering spectrum (Lorentzian) to give

$$\rho(\epsilon) = \frac{1}{1 + \left(\frac{\epsilon - \epsilon_R}{\Gamma/2}\right)^2}, \tag{22}$$

where ϵ_R is the peak wavelength and Γ is the FWHM.

The spectra must be corrected for the quantum efficiency (QE) of the spectrometer detector. Values for the QE are given by the manufacturer of the spectrometer which can be numerically fit with an nth order polynomial to good accuracy. Thus, the corrected linear emission spectrum is

$$\phi_{\text{corr}}(\epsilon) = \phi_{\text{Th+NF}}(\epsilon) \times \rho(\epsilon) \times \text{QE}(\epsilon) \tag{23}$$

This calculated linear spectrum shown is in Supplementary Figure 7.

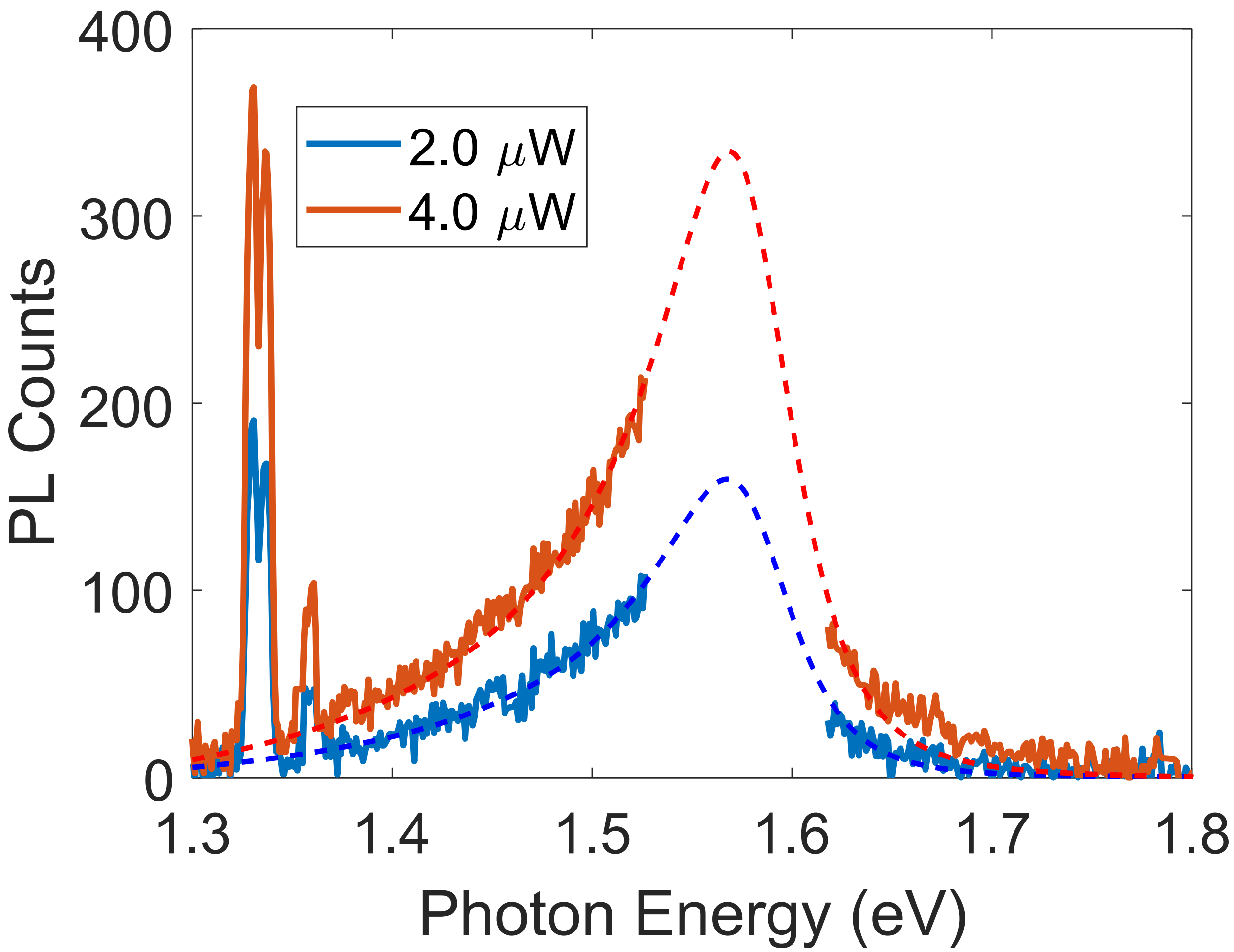

Supplementary Figure 7: **Linear spectra fitting.** 2 nm PL spectra fitted to calculated non-Fermi/thermal linear spectra. Fitting parameters for $\Delta\rho^0_{\text{nf}}$ and T_{eff} are the corresponding fit parameters given in Table S1; fixed gap and resonance varied fluence ranges; $2.0 - 4.6$ μJ

References

(1) Roloff L., Klemm P., Gronwald I., Huber R., Lupton J. M.& Bange S. Light emission from gold nanoparticles under ultrafast near-infrared excitation: Thermal radiation, inelastic light scattering, or multiphoton luminescence? *Nano Letters* **17**, 7914–7919 (2017).

(2) Novotny, L. & Hecht, B. *Principles of nano-optics.* (Cambridge University Press, Cambridge, 2012).

(3) Efrima, S. & Metiu, H. Classical theory of light scattering by an adsorbed molecule. I. Theory. *J. Chem. Phys.* **70**, 1602–1613 (2018).

(4) Carles, R., Bayle, M., Benzo, P., Benassayag, G., Bonafos, C., Cacciato, G. & Privitera, V. Plasmon-resonant Raman spectroscopy in metallic nanoparticles: Surface-enhanced scattering by electronic excitations. *Physical Review B* **92**, (2015).

(5) Zawadowski, A. & Cardona, M. Theory of raman scattering on normal metals with impurities. *Physical Review B* **42**, 10732–10734 (1990).

(6) Haug T., Klemm P., Bange S.& Lupton J. M. Hot-electron intraband luminescence from single hot spots in noble-metal nanoparticle films. *Phys. Rev. Lett.* **115**, 067403 (2015).

(7) Boyd, G. T., Yu, Z. H. & Shen, Y. R. Photoinduced luminescence from the noble metals and its enhancement on roughened surfaces. *Physical Review B* **33**, 7923–7936 (1986).
